# Relationship between labral length and symptoms in patients with acetabular dysplasia before rotational acetabular osteotomy

**DOI:** 10.1093/jhps/hnac045

**Published:** 2022-10-17

**Authors:** Yuichi Shirogane, Yasuhiro Homma, Naotake Yanagisawa, Masanori Higano, Yoichiro Hirasawa, Shigeru Nakamura, Tomonori Baba, Kazuo Kaneko, Hitoshi Taneda, Muneaki Ishijima

**Affiliations:** Department of Orthopaedic Surgery, Nishitokyo Chuo General Hospital, 2-4-19 Shibakubocho, Nishitokyo-shi, Tokyo 188-0014,Japan; Department of Medicine for Orthopaedics and Motor Organ, Juntendo University Graduate School of Medicine, 2-1-1 Hongo, Bunkyo-ku, Tokyo 133-8421, Japan; Department of Orthopaedic, Faculty of Medicine, Juntendo University, 3-1-3 Hongo, Bunkyo-ku, Tokyo 133-8431, Japan; Department of Orthopaedic Surgery, Nishitokyo Chuo General Hospital, 2-4-19 Shibakubocho, Nishitokyo-shi, Tokyo 188-0014,Japan; Department of Medicine for Orthopaedics and Motor Organ, Juntendo University Graduate School of Medicine, 2-1-1 Hongo, Bunkyo-ku, Tokyo 133-8421, Japan; Department of Orthopaedic, Faculty of Medicine, Juntendo University, 3-1-3 Hongo, Bunkyo-ku, Tokyo 133-8431, Japan; Clinical Research and Trial Center, Juntendo University, 3-1-3 Hongo, Bunkyo-ku, Tokyo 133-8431, Japan; Department of Orthopaedic Surgery, Nishitokyo Chuo General Hospital, 2-4-19 Shibakubocho, Nishitokyo-shi, Tokyo 188-0014,Japan; Department of Orthopaedic Surgery, Nishitokyo Chuo General Hospital, 2-4-19 Shibakubocho, Nishitokyo-shi, Tokyo 188-0014,Japan; Department of Orthopaedic Surgery, Nishitokyo Chuo General Hospital, 2-4-19 Shibakubocho, Nishitokyo-shi, Tokyo 188-0014,Japan; Department of Medicine for Orthopaedics and Motor Organ, Juntendo University Graduate School of Medicine, 2-1-1 Hongo, Bunkyo-ku, Tokyo 133-8421, Japan; Department of Orthopaedic, Faculty of Medicine, Juntendo University, 3-1-3 Hongo, Bunkyo-ku, Tokyo 133-8431, Japan; Department of Medicine for Orthopaedics and Motor Organ, Juntendo University Graduate School of Medicine, 2-1-1 Hongo, Bunkyo-ku, Tokyo 133-8421, Japan; Department of Orthopaedic, Faculty of Medicine, Juntendo University, 3-1-3 Hongo, Bunkyo-ku, Tokyo 133-8431, Japan; Department of Orthopaedic Surgery, Nishitokyo Chuo General Hospital, 2-4-19 Shibakubocho, Nishitokyo-shi, Tokyo 188-0014,Japan; Department of Medicine for Orthopaedics and Motor Organ, Juntendo University Graduate School of Medicine, 2-1-1 Hongo, Bunkyo-ku, Tokyo 133-8421, Japan; Department of Orthopaedic, Faculty of Medicine, Juntendo University, 3-1-3 Hongo, Bunkyo-ku, Tokyo 133-8431, Japan

## Abstract

The aim of this study was to investigate the relationship between acetabular labral length and symptoms in patients with acetabular dysplasia. In a retrospective medical record review, 218 patients with acetabular dysplasia who had undergone rotational acetabular osteotomy were identified. After implementing the inclusion and exclusion criteria, 53 patients were analyzed for preoperative symptoms measured by the Japanese Orthopaedic Association Hip Disease Evaluation Questionnaire (JHEQ), acetabular bone morphology parameters by anteroposterior pelvic radiographs and labral parameters by radial magnetic resonance imaging. Spearman’s correlation coefficients were calculated among JHEQ scores, bone morphologic parameters and labral parameters. Multiple linear regression models to determine the predictive variables of JHEQ score and labral length were obtained. There was no correlation between bone morphologic parameters and JHEQ scores. Labral length measured anteriorly correlated with JHEQ pain {*r* [95% confidence interval (CI)] = −0.335 (−0.555, −0.071), *P* = 0.014}, movement subscale [*r* (95% CI) = −0.398 (−0.603, −0.143), *P* = 0.003], mental subscale [*r* (95% CI) = −0.436 (−0.632, −0.188), *P* = 0.001] and total JHEQ score [*r* (95% CI) = −0.451 (−0.642, −0.204), *P* = 0.001]. The multiple linear regression results showed that anterior labral length was independently associated with JHEQ subscales in some models. Meanwhile, age, acetabular head index and total JHEQ score were independently associated with anterior labral length in all models. Labral length, notably in anterosuperior area, in patients with symptomatic acetabular dysplasia was related to patient’s symptom. Labral length may be an important objective image finding that can be used to assess the severity of cumulative hip instability.

## INTRODUCTION

The main pathophysiology of acetabular dysplasia is thought to be instability of the femoral head in the acetabulum [1]. Although acetabular bone coverage is the main stabilizer for the hip [2], soft tissue stabilizers such as the labrum [3], capsular ligament [4, 5], ligamentum teres [6] and surrounding muscles [7] are also important [8]. Degeneration of the acetabular labrum has become a widely recognized cause of hip pain [9–12]. Kim *et al*. confirmed the presence of nerve endings in the acetabular labrum [11], and Klaue *et al*. reported labral damage in patients with symptomatic acetabular dysplasia without advanced arthritic changes [13].

Radial array magnetic resonance imaging (MRI) is a relatively new technique that is effective for delineating most of the acetabular labrum. Because the labrum is annularly attached to the acetabular rim, it is difficult to visualize in conventional axial, coronal and sagittal MRI views. However, radial array MRI can obtain oblique images through the anterior and posterior edges of the acetabulum from a localizer through the center of the femoral head, which is used as a base axis to obtain radial cross-sectional images [14].

Recent studies have characterized the acetabular labrum in detail and have shown its importance in acetabular dysplasia. Ueshima *et al*. reported that staging using radial MRI is superior to center–edge angle (CEA) evaluation for predicting the progression of joint narrowing in patients with acetabular dysplasia [15]. Moreover, previous studies have demonstrated a relationship between labrum size and acetabular bone morphology. Kuroda *et al*. found that labrum length increases when the acetabulum is hypoplastic [16]. In patients with normal CEA and those with acetabular dysplasia, Horii *et al*. reported anterior labrum lengths of 8.7 and 14.3 mm, respectively [9]. Although previous studies have clarified the morphological features of the acetabular labrum in acetabular dysplasia, the clinical importance of these morphological changes has not been clearly explained. In particular, the relationship between acetabular labrum length and patient symptoms remains unknown. Moreover, an objective evaluation for symptomatic acetabular dysplasia is scarce. Thereby, it is necessary to establish the objective evaluation in image findings for an assessment of symptomatic acetabular dysplasia. The aim of this study was to investigate the relationship between acetabular labrum length and patient’s symptom.

## MATERIALS AND METHODS

### Patients (Fig. 1)

**Fig. 1.**
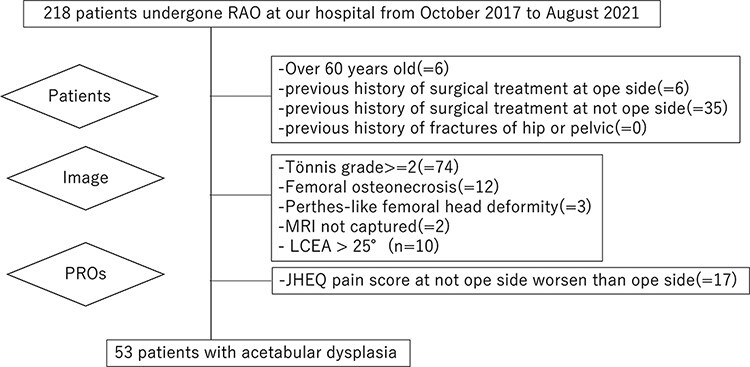
Study flowchart.

In a retrospective medical record review, we identified 218 patients who had undergone rotational acetabular osteotomy (RAO) for symptomatic acetabular dysplasia at our hospital from October 2017 to August 2021 (Fig. 1). In order to minimize the degenerative effect and focus on the hip instability by symptomatic acetabular dysplasia, exclusion criteria were set as follows: (i) age >60 years (*n* = 6), (ii) femoral head osteonecrosis (*n* = 12), (iii) previous history of surgical treatment of acetabular dysplasia (*n* = 41), (iv) Tönnis grade 2 or higher (*n* = 74), (v) Perthes-like femoral head deformity (*n* = 3), (vi) previous history of hip or pelvic fracture (*n* = 0), (vii) lateral CEA (LCEA) >25° (*n* = 10) and (viii) acetabular retroversion (*n* = 0). We also excluded patients who did not undergo MRI (*n* = 2) and those with a worse Japanese Orthopaedic Association Hip Disease Evaluation Questionnaire (JHEQ) score on the nonoperative side than the operative side (*n* = 17). Finally, 53 patients (1 man and 52 women) were included for analysis. Institutional ethics board approval was obtained.

### Outcomes

Patient’s symptom was measured using the JHEQ before RAO. The JHEQ is a highly reliable self-administered outcome evaluation tool used in patients with hip joint disease, taking into account the existing quality of life evaluation criteria such as the Medical Outcome Study 36-Item Short-Form Hearth Survey (SF-36) and facets of the Asian lifestyle [17] that consists of pain (28 points), movement (28 points) and mental (28 points) subscales (maximum score, 84 points); higher scores indicate a better outcome. Each questionnaire item is scored between 0 and 4 points.

### Radiographic evaluation

The following bone morphologic parameters were obtained on standard anteroposterior pelvic radiographs:

- LCEA: the angle between the perpendicular line of the center of the femoral head and the lateral aspect of the sourcil [16, 18].
- Acetabular head index (AHI): calculated as the ratio of the distance between the medial tip of the femoral head and the lateral edge of the acetabular roof to the size of the femoral head [19].
- Sharp angle: defined as the angle between the lower edge of the pelvic teardrop and the line connecting the lower edge of the teardrop and the outer edge of the acetabulum [20].
- Acetabular roof obliquity (ARO): defined as the angle between a line connecting the lateral edge of the acetabular roof and the inferior edge of the sourcil and a line parallel to both pelvic tear drops [16, 21].
- Femoro-epiphyseal acetabular roof (FEAR) index: defined as the angle between the physeal scar of the femoral head and sourcil and considered a measurement associated with instability in patients with developmental dysplasia of the hip (DDH) [16, 22].

### MRI (Fig. 2)

Patients were evaluated before RAO using a standard orthopedic MRI hip protocol on a 1.5T system (GE Healthcare, Chicago, IL, USA) with a phased array torso coil from the superior edge of the pelvis to just below the proximal femur. The MRI hip protocol consisted of an axial T1-weighted sequence, an axial T2-weighted sequence and coronal T1-weighted and T2-weighted sequences. A slice thickness of 5 mm and a gap of 1.0 mm were used. The field of view was 38 cm with a matrix size of 416 × 256. Radially sectioned images passing through the center of the femoral head every 15° from 90° anterior to 90° posterior were obtained.

**Fig. 2.**
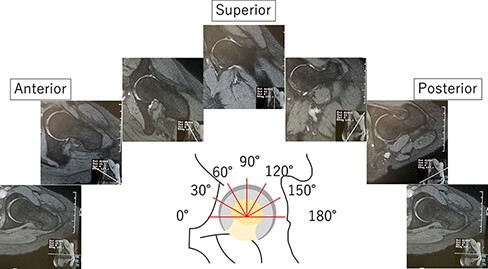
Positions used to measure the labral parameter. Position of each of the seven radial slices (0°, 30°, 60°, 90°, 120°, 150° and 180°). Anterior is indicated by the 0°, 30° and 60° slices. Superior is indicated by the 60°, 90° and 120° slices. Posterior is indicated by the 120°, 150° and 180° slices.

### Labral length and index (Fig. 3)

**Fig. 3.**
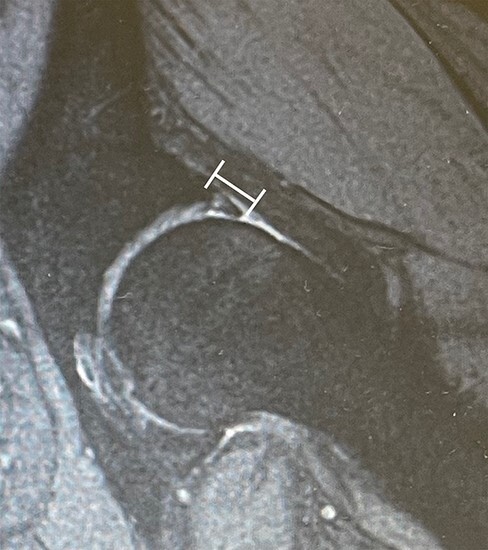
Measurement to labral parameter. Measurement as the distance from the acetabular rim to the edge of the labral length at each slice of the radial MRI scan (white line).

Labral length was measured at seven anatomical sites along the acetabular rim: every 30° from 0° to 180° (Fig. 2) [16, 21]. All measurements were obtained by a single orthopedist well-versed in radial hip MRI who was blinded to other data. The labral length was measured from the acetabular rim to the free end of the labrum (in mm) using a Digital Imaging and Communications in Medicine picture archiving and communications systems workstation (Secure DICOM Server viewer; NOBORI, Tokyo, Japan) (Fig. 3). In a previous article, high inter-observer reliability and intra-observer reliability (0.86 and 0.89, respectively) for labral length measurements were reported [21]. Zero degrees to 60° was considered anterior, 60° to 120° was considered superior and 120° to 180° was considered posterior. Labral index was defined as the length of the labrum divided by the radius of the femoral head, as previously described [23].

### Statistical analysis

Statistical analyses were performed using SPSS software version 27 (IBM, Armonk, NY, USA). Data were tested for normality using the Shapiro–Wilk test. Medians with interquartile range were calculated for each variable examined. Spearman correlation coefficients were calculated between JHEQ scores, bone morphologic parameters and labrum parameters. The following correlation coefficient (*r*) scale was used to describe the correlation strength: very strong, 0.80 ≤ *r* ≤ 1.0; strong, 0.60 ≤ *r* ≤ 0.79; moderate, 0.4 ≤ *r* ≤ 0.59; weak, 0.39 ≤ *r* ≤ 0.2 and very weak, 0.19 ≤ *r* ≤ 0.00. *P* ≤ 0.05 was considered significant [16, 24]. In the multiple regression analyses, the variables were selected from the categories of patient characteristics, bone morphologic parameters and labral length with the lowest *P*-value in univariate analysis and without a correlation coefficient of |*r*| > 0.8 between the independent variables. 95% CIs for correlation coefficients were calculated using the bootstrap method. From these variables, we used multiple regression analysis models to determine which variables were predictors for the JHEQ pain subscale, JHEQ total score, and anterior labral length. Multiple regression was performed using the forced entry method.

All measurements were carried out by an orthopedic surgeon (Y.S.); measurements were performed twice, and intra-observer reliability was calculated to examine the reproducibility of measurements with an intraclass correlation coefficient (ICC) using a subset of all cases. The intra-observer reliability was evaluated with a one-way random effects and single measures model.

The power (1-β) was calculated. The conditions were as follows; spearman correlation between anterior labral length and each JHEQ scores; alpha = 0.05, sample size = 53.

## RESULTS

### Participants and descriptive data

Patient characteristics and bone morphologic parameters are shown in Table I.

**Table I.** Patient characteristics and radiographic parameters

	Number of patients		53
	Preoperative factors		
	Sex (male/female)		1/52
	Age		37 (16.5) years old
	Height		159 (6) cm
	Weight		54.4 (11) kg
	BMI		22.8 (3.773)
	JHEQ subscale score		
	Pain		10 (10.5)
	Movement		16 (16)
	Mental		13 (11.5)
	Total		38 (29.5)
	Bone morphological parameters		
	LCEA		15.0 (9.5)°
	AHI		0.692 (0.074)
	Sharp angle		47.0 (3.5)°
	ARO		17.4 (8.6)°
	FEAR index		−5.8 (11.9)
	Soft tissue parameters		(mm)
		0°	9.9 (2.5)
		30°	9.3 (3.2)
		60°	10.1 (3.9)
Labral length		90°	11.0 (3.6)
		120°	11.3 (3.4)
		150°	10.9 (2.4)
		180°	9.65 (2.5)
		0°	0.46 (0.123)
		30°	0.44 (0.149)
		60°	0.47 (0.144)
Labral index^a^		90°	0.54 (0.148)
		120°	0.53 (0.147)
		150°	0.51 (0.115)
		180°	0.46 (0.101)

Data are presented as medians with interquartile range.

a Labral length/femoral head radius.

### Intra-observer reliability and power analysis

The ICC (intra) was >0.8 for all measurements (LCEA: 0.895, AHI: 0.904, sharp angle: 0.880, ARO: 0.926, FEAR index: 0.892 and labral length: 0.808). These ICCs (intra-observer reliability) were similar to previous reports [16, 21, 25]. Based on the reliability observed above, the means of the two measurements were used for all analyses.

A power analysis was performed between anterior labral length and JHEQ scores with alpha 0.05, sample size 53 and each correlation coefficient. The results demonstrated adequate power in each (total JHEQ score: 0.916. JHEQ-pain: 0.668, JHEQ-movement: 0.825, JHEQ-mental: 0.894).

### Correlation among bone morphologic parameters, labral parameters and JHEQ score

There was no correlation between the bone morphologic parameters and JHEQ scores (Table II). The total sum of labral lengths measured at all seven sites significantly correlated with pain subscale [*r* (95% CI) = −0.328 (−0.550, −0.063), *P* = 0.016], movement subscale [*r* (95% CI) = −0.363 (−0.576, −0.102), *P* = 0.008], mental subscale [*r* (95% CI) = −0.364 (−0.578, −0.104), *P* = 0.007] and total JHEQ score [*r* (95% CI) = −0.406 (−0.609, −0.152), *P* = 0.003]. Labral length measured anteriorly (sum of 0° to 60°) significantly correlated with pain subscale [*r* (95% CI) = −0.335 (−0.555, −0.071), *P* = 0.014], movement subscale [*r* (95% CI) = −0.398 (−0.603, −0.143), *P* = 0.003], mental subscale [*r* (95% CI) = −0.436 (−0.632, −0.188), *P* = 0.001] and total JHEQ score [*r* (95% CI) = −0.451 (−0.642, −0.204), *P* = 0.001]. At zero, labral length significantly correlated with pain subscale [*r* (95% CI) = −0.350 (−0.567, −0.089), *P* = 0.010], movement subscale [*r* (95% CI) = −0.324 (−0.547, −0.060), *P* = 0.018], mental subscale [*r* (95% CI) = −0.390 (−0.597, −0.133), *P* = 0.004] and total JHEQ score [*r* (95% CI) = −0.408 (−0.611, −0.156), *P* = 0.002]. On the other hand, the anterior labral index, corrected for labral length by the radius of the femoral head, was significantly correlated with pain [*r* (95% CI) = −0.306 (−0.532, −0.039), *P* = 0.026] and total JHEQ score [*r* (95% CI) = −0.299 (−0.501, −0.108), *P* = 0.029], which were lower than labral length. Tables III and IV show the correlations of labral length and index with JHEQ subscales and total JHEQ scores.

**Table II.** Correlation analysis of bone morphologic parameters and JHEQ scores

	*Pain*		*Movement*		*Mental*		*Total*	
	*r (95% CI)*	*P-value*	*r (95% CI)*	*P-value*	*r (95% CI)*	*P-value*	*r (95% CI)*	*P-value*
LCEA	0.119 (−0.156, 0.377)	0.395	0.091 (−0.184, 0.353)	0.517	−0.151 (−0.405, 0.125)	0.281	0.033 (−0.239, 0.301)	0.814
AHI	0.077 (−0.197, 0.341)	0.582	0.117 (−0.158, −0.375)	0.404	−0.060 (−0.325, 0.214)	0.669	0.072 (−0.203, 0.335)	0.610
Sharp angle	−0.034 (−0.301, 0.239)	0.808	−0.087 (−0.188, 0.349)	0.537	0.185 (−0.090, 0.434)	0.185	0.085 (−0.189, 0.348)	0.543
ARO	−0.036 (−0.304, 0.236)	0.796	−0.061 (−0.326, 0.213)	0.666	0.010 (−0.261, 0.280)	0.942	−0.029 (−0.297, 0.243)	0.835
FEAR index	−0.045 (−0.228, 0.311)	0.767	−0.078 (−0.197, 0.341)	0.607	0.098 (−0.177, 0.359)	0.517	0.112 (−0.163, 0.371)	0.459

*P* < 0.05 is considered significant.

**Table III.** Correlation analysis of labral length and JHEQ scores

	*Pain*		*Movement*		*Mental*		*Total*	
*Labral length*	*r (95% CI)*	*P-value*	*r (95% CI)*	*P-value*	*r (95% CI)*	*P-value*	*r (95% CI)*	*P-value*
0°	−0.350 (−0.567, −0.089)	0.010*	−0.324 (−0.547, −0.060)	0.018*	−0.390 (−0.597, −0.133)	0.004*	−0.408 (−0.611, −0.156)	0.002*
30°	−0.315 (−0.540, −0.050)	0.021*	−0.410 (−0.613, −0.158)	0.002*	−0.389 (−0.597, −0.133)	0.004*	−0.428 (−0.627, −0.180)	0.001*
60°	−0.236 (−0.476, 0.036)	0.089	−0.302 (−0.529, 0.035)	0.028*	−0.381 (−0.591, −0.125)	0.005*	−0.353 (−0.570, −0.092)	0.009*
90°	−0.333 (−0.553, −0.068)	0.015*	−0.364 (−0.577, −0.103)	0.007*	−0.320 (−0.543, −0.055)	0.019*	−0.389 (−0.597, −0.0133)	0.004*
120°	−0.213 (−0.462, 0.054)	0.126	−0.213 (−0.441, 0.08)	0.126	−0.212 (−0.455, 0.063)	0.128	−0.240 (−0.479, 0.033)	0.083
150°	−0.198 (−0.444, 0.076)	0.154	−0.237 (−0.478, 0.034)	0.088	−0.183 (−0.434, 0.090)	0.189	−0.241 (−0.481, 0.030)	0.082
180°	−0.209 (−0.451, 0.068)	0.133	−0.217 (−0.460, 0.057)	0.119	−0.180 (−0.431, 0.093)	0.196	−0.251 (−0.487, 0.022)	0.070
Anterior	−0.335 (−0.555, −0.071)	0.014*	−0.398 (−0.603, −0.143)	0.003*	−0.436 (−0.632, −0.188)	0.001*	−0.451 (−0.642, −0.204)	0.001*
Superior	−0.272 (−0.505, 0.002)	0.049*	−0.318 (−0.542, −0.052)	0.021*	−0.326 (−0.549, −0.062)	0.017*	−0.347 (−0.565, −0.086)	0.011*
Anterior + superior	−0.343 (−0.562, −0.081)	0.012*	−0.379 (−0.589, −0.121)	0.005*	−0.393 (−0.600, −0.138)	0.004*	−0.424 (−0.623, −0.173)	0.002*
Posterior	−0.232 (−0.474, 0.039)	0.095	−0.269 (−0.504, 0.000)	0.051	−0.242 (−0.480, 0.031)	0.080	−0.292 (−0.521, −0.024)	0.034*
Total	−0.328 (−0.550, −0.063)	0.016*	−0.363 (−0.576, −0.102)	0.008*	−0.364 (−0.578, 0.104)	0.007*	−0.406 (−0.609, −0.152)	0.003*

* indicates significance (*P* < 0.05).

Anterior, sum of 0° to 60°; superior, sum of 60° to 120° and posterior, sum of 120° to 180.

**Table IV.** Correlation analysis of labral index and JHEQ scores

	*Pain*		*Movement*		*Mental*		*Total*	
*Labral index*	*r (95% CI)*	*P-value*	*r (95% CI)*	*P-value*	*r (95% CI)*	*P-value*	*r (95% CI)*	*P-value*
0°	−0.340 (−0.559, −0.077)	0.013*	−0.271 (−0.504, 0.001)	0.050*	−0.360 (−0.574, −0.099)	0.008*	−0.358 (−0.573, −0.098)	0.008*
30°	−0.313 (−0.538, −0.047)	0.022*	−0.369 (−0.582, −0.110)	0.006*	−0.409(−0.612, 0.156)	0.002*	−0.410 (−0.613, 0.158)	0.002*
60°	−0.174 (−0.424, 0.101)	0.213	−0.242 (−0.481, 0.030)	0.081	−0.365 (−0.578, −0.105)	0.007*	−0.290 (−0.520, 0.022)	0.035*
90°	−0.278 (−0.510, 0.008)	0.044*	−0.271 (−0.504, −0.001)	0.050*	−0.254 (−0.491, 0.018)	0.067	−0.292 (−0.521, −0.024)	0.034*
120°	−0.153 (−0.407, 0.122)	0.273	−0.128 (−0.385, 0.147)	0.361	−0.132 (−0.389, 0.143)	0.346	−0.144 (−0.399, 0.131)	0.302
150°	−0.168 (−0.420, 0.107)	0.228	−0.127 (−0.384, 0.148)	0.365	−0.085 (−0.348, 0.189)	0.543	−0.135 (−0.391, 0.140)	0.335
180°	−0.161 (−0.413, 0.114)	0.250	−0.121 (−0.379, 0.154)	0.388	−0.122 (−0.380, 0.154)	0.385	−0.160 (−0.413, 0.115)	0.252
Anterior	−0.306 (−0.532, −0.039)	0.026*	−0.351 (−0.567, −0.089)	0.010*	−0.430 (−0.627, −0.180)	0.001*	−0.406 (−0.609, −0.153)	0.003*
Superior	−0.239 (−0.478, 0.034)	0.085	−0.259 (−0.495, 0.012)	0.061	−0.304 (−0.531, −0.037)	0.027*	−0.293 (−0.522, 0.024)	0.033*
Anterior + superior	−0.315 (−0.539, −0.049)	0.022*	−0.312 (−0.537, −0.045)	0.023*	−0.361 (−0.575, −0.101)	0.008*	−0.363 (−0.577, −0.103)	0.008*
Posterior	−0.195 (−0.442, 0.080)	0.163	−0.151 (−0.405, 0.1324)	0.280	−0.135 (−0.391, 0.141)	0.336	−0.179 (−0.428, 0.096)	0.201
Total	−0.284 (−0.515, −0.015)	0.039*	−0.268 (−0.502, 0.003)	0.053	−0.310 (−0.535, −0.043)	0.024*	−0.316 (−0.540, −0.050)	0.021*

* indicates significance (*P* < 0.05).

Anterior, sum of 0° to 60°; superior, sum of 60° to 120° and posterior, sum of 120° to 180.

Table V shows the correlations of labral length and index with bone morphologic parameters. Labral length was significantly and moderately correlated with LCEA, ARO, AHI and FEAR index in each compartment. In the anterior labrum length, weak correlations were found for LCEA [*r* (95% CI) = −0.298 (−0.526, −0.030), *P* = 0.0230], AHI [*r* (95% CI) = −0.287 (−0.516, −0.017), *P* = 0.037], ARO [*r* (95% CI) = 0.384 (0.128, −0.593), *P* = 0.004] and FEAR index [*r* (95% CI) = 0.281 (0.011, −0.512), *P* = 0.059]. Total labral length was moderately correlated with LCEA [*r* (95% CI) = −0.446 (−0.639, −0.200), *P* = 0.001], AHI [*r* (95% CI) = −0.382 (−0.591, −0.124), *P* = 0.005] and ARO [*r* (95% CI) = 0.486 (−0.249, −0.669), *P* < 0.0001] and FEAR index [*r* (95% CI) = 0.400 (0.146, −0.605), *P* = 0.006]. Labrum index was significantly and moderately correlated.

**Table V.** Correlation analysis of bone morphologic parameters and labral measurements

	*LCEA*		*AHI*		*Sharp angle*		*ARO*		*FEAR index*	
	*r (95% CI)*	*P-value*	*r (95% CI)*	*P-value*	*r (95% CI)*	*P-value*	*r (95% CI)*	*P-value*	*r (95% CI)*	*P-value*
Labral length										
Anterior	−0.298 (−0.526, −0.030)	0.0230*	−0.287 (−0.516, −0.017)	0.037*	0.081 (−0.194, 0.344)	0.565	0.384 (0.128, 0.593)	0.004*	−0.281 (0.011, 0.512)	0.059
Superior	−0.431 (−0.628, −0.181)	0.001*	−0.380 (−0.589, −0.121)	0.005*	0.144 (−0.134, 0.396)	0.304	0.423 (0.173, 0.622)	0.002*	0.425 (0.175, 0.624)	0.003*
Anterior + superior	−0.392 (−0.599, −0.136)	0.004*	−0.341 (−0.560, −0.078)	0.012*	0.133 (−0.44, 0.388)	0.343	0.447 (0.200, 0.640)	0.001*	0.413 (0.160, 0.614)	0.004*
Posterior	−0.470 (−0.657, −0.229)	0.0001*	−0.396 (−0.602, −0.141)	0.003*	0.292 (−0.022, 0.520)	0.034*	0.488 (0.251, 0.670)	0.0001*	0.383 (0.127, 0.593)	0.009*
Total	−0.446 (−0.639, −0.200)	0.001*	−0.382 (−0.591, −0.124)	0.005*	0.210 (−0.065, 0.453)	0.131	0.486 (0.249, 0.669)	0.0001*	0.400 (0.146, 0.605)	0.006*
Labral index										
Anterior	−0.332 (−0.553, −0.068)	0.015*	−0.299 (−0.526, −0.031)	0.030*	0.076 (−0.198, 0.340)	0.588	0.329 (0.064, 0.550)	0.016*	−0.266 (−0.005, 0.500)	0.074
Superior	−0.490 (−0.671, −0.253)	0.0001*	−0.407 (−0.610, −0.154)	0.002*	0.178 (−0.098, 0.427)	0.203	0.389 (0.132, 0.596)	0.004*	0.403 (−0.148, 0.607)	0.006*
Anterior + Superior	−0.459 (−0.6549, −0.216)	0.001*	−0.379 (−0.589, −0.121)	0.005*	0.176 (−0.100, 0.425)	0.209	0.394 (0.138, 0.600)	0.004*	0.379 (−0.121, 0.589)	0.009*
Posterior	−0.511 (−0.687, −0.280)	0.0001*	−0.434 (−0.631, −0.186)	0.001*	0.331 (0.066, 0.551)	0.015*	0.449 (0.204, 0.642)	0.001*	0.407 (−0.153, 0.610)	0.005*
Total	−0.486 (−0.669, −0.248)	0.0001*	−0.390 (−0.597, −0.134)	0.004*	0.230 (−0.044, 0.470)	0.097	0.448 (0.202, 0.640)	0.001*	0.382 (0.124, 0.591)	0.009*

* indicates significance (*P* < 0.05).

### Independent predictors of JHEQ pain subscale score

Table VI (Column 1) shows the univariate and multivariate linear regression analyses of patient characteristics, bone morphologic parameters and labral length, with JHEQ pain subscale as the dependent variable. According to the variable selection method as described before, body mass index (BMI), LCEA and anterior labral length were selected for multivariate analysis (BMI: *P* = 0.185, LCEA: *P* = 0.249 and anterior labral length: *P* = 0.014) and three models with different variables were tested. The results showed that anterior labral length was independently associated with JHEQ pain subscales in three models (Model 1: β coefficient = −0.318 and adjusted *R*^2^ = 0.095. Model 2: β coefficient = −0.322 and adjusted *R*^2^ = 0.078. Model 3: β coefficient = −0.299 and adjusted *R*^2^ = 0.078).

**Table VI.** Univariate and multivariate regression analyses for JHEQ pain subscale

	*Univariate analysis*		*Multivariate analysis*					
			*Model 1*		*Model 2*		*Model 3*	
			*Adjusted R^2^ = 0.095*		*Adjusted R^2^ = 0.78*		*Adjusted R^2^ = 0.078*	
	*β (95% CI)*	*P-value*	*β (95% CI)*	*P-value*	*β (95% CI)*	*P-value*	*β (95% CI)*	*P-value*
Patient characteristics								
Age	−0.106 (−0.229, 0.103)	0.452	–	–	–	–	–	–
Height	0.073 (−0.250, 0.426)	0.603	–	–	–	–	–	–
Weight	−0.138 (−0.253, 0.085)	0.324	–	–	–	–	–	–
BMI	−0.185 (−0.804, 0.159)	0.185	−0.135 (−0.703, 0.233)	0.318	–	–	−0.135 (−0.709, 0.236)	0.320
Bone morphologic parameters								
LCEA	0.161 (−0.111, 0.421)	0.249	–	–	0.034 (−0.247, −0.312)	0.817	0.036 (−0.245, 0.314)	0.804
AHI	0.132 (−12.769, 35.699)	0.347	–	–	–	–	–	–
Sharp angle	−0.074 (−0.725, 0.423)	0.599	–	–	–	–	–	–
ARO	−0.013 (−0.295, 0.268)	0.924	–	–	–	–	–	–
FEAR index	0.029 (−0.233, 0.281)	0.850	–	–	–	–	–	–
Labral length								
Anterior	−0.335 (−0.623, −0.073)	0.014*	−0.314 (−0.604, −0.047)	0.023*	−0.322 (−0.636, −0.032)	0.031*	−0.299 (−0.617, −0.004)	0.047*
Superior	−0.278 (−0.556, −0.008)	0.044*	–	–	–	–	–	–
Superior + anterior	−0.330 (−0.389, −0.042)	0.016*	–	–	–	–	–	–
Posterior	−0.226 (−0.574, 0.055)	0.104	–	–	–	–	–	–
Total	−0.320 (−0.298, −0.027)	0.020*	–	–	–	–	–	–

Model 1 = AHI and anterior labral length.

Model 2 = Age, AHI and anterior labral length.

Model 3 = Age, BMI, AHI and anterior labral length.

* Indicates significance (*P* < 0.05).

### Independent predictors of total JHEQ score

Table VII shows the univariate and multivariate linear regression analyses with total JHEQ score as the dependent variable. Age, BMI, AHI and anterior labral length were selected for multivariate analysis (age: *P* = 0.004, BMI: *P* = 0.071, AHI: *P* = 0.563 and anterior labral length: *P* = 0.001), and three models with different variables were tested. The results showed that anterior labral length was independently associated with total JHEQ score in three models (Model 1: β coefficient = −0.335 and adjusted *R*^2^ = 0.224. Model 2: β coefficient = −0.334 and adjusted *R*^2^ = 0.208. Model 3: β coefficient = −0.299 and adjusted *R*^2^ = 0.250). Age was also independently associated with total JHEQ score in three models (Model 1: β coefficient = −0.281 and adjusted *R*^2^ = 0.224. Model 2: β coefficient = −0.281 and adjusted *R*^2^ = 0.208. Model 3: β coefficient = −0.292 and adjusted *R*^2^ = 0.250).

**Table VII.** Univariate and multivariate regression analyses for total JHEQ score

	*Univariate analysis*		*Multivariate analysis*					
			*Model 1*		*Model 2*		*Model 3*	
			*Adjusted R^2^ = 0.224*		*Adjusted R^2^ = 0.208*		*Adjusted R^2^ = 0.250*	
	*β (95% CI)*	*P-value*	*β (95% CI)*	*P-value*	*β (95% CI)*	*P-value*	*β (95% CI)*	*P-value*
Patient characteristics								
Age	−0.392 (−1.137, −0.234)	0.004*	−0.281 (−0.945, −0.036)	0.035*	−0.281 (−0.987, 0.003)	0.026*	−0.292 (−0.958, −0.063)	0.026*
Height	−0.098 (−1.340, 0.643)	0.483	–	–	–	–	–	–
Weight	−0.256 (−0.942, 0.029)	0.064	–	–	–	–	–	–
BMI	−0.250 (−2.679, 0.113)	0.071	–	–	–	–	−0.201 (−2.289, 0.222)	0.105
Bone morphologic parameters								
LCEA	0.062 (−0.615, 0.967)	0.657	–	–	–	–	–	–
AHI	0.081 (−50.910, 92.459)	0.563	–	–	0.002 (−74.026, 74.923)	0.990	–	–
Sharp angle	0.053 (−1.374, 2.007)	0.708	–	–	–	–	–	–
ARO	0.006 (−0.810, 0.846)	0.966	–	–	–	–	–	–
FEAR index	0.068 (−0.602, 0.953)	0.651	–	–	–	–	–	–
Labral length								
Anterior	−0.428 (−2.083, −0.533)	0.001*	−0.335 (−1.817, −0.229)	0.013*	−0.334 (−1.9145, 0.095)	0.031*	−0.299 (−1.706, −0.121)	0.025*
Superior	−0.357 (−1.850, −0.283)	0.009*	–	–	–	–	–	–
Superior + anterior	−0.407 (−1.274, −0.289)	0.002*	–	–	–	–	–	–
Posterior	−0.245 (−1.749, 0.093)	0.077	–	–	–	–	–	–
Total	−0.385 (−0.963, −0.187)	0.004*	–	–	–	–	–	–

Model 1 = Age and anterior labral length.

Model 2 = Age, AHI and anterior labral length.

Model 3 = Age, BMI and anterior labral length.

* Indicates significance (*P* < 0.05).

### Independent predictors of anterior labral length

Table VIII shows the univariate and multivariate linear regression analyses with anterior labral length as the dependent variable. Age, AHI and total JHEQ score were selected for multivariate analysis as the variables with the lowest *P*-values from each of patient characteristics, bone morphologic parameters and JHEQ score categories (Age: *P* = 0.015, AHI: *P* = 0.003 and total: *P* = 0.001). The results showed that AHI were independently associated with anterior labral length in three models (Model 1: β coefficient = −0.478 and adjusted *R*^2^ = 0.303. Model 2: β coefficient = −0.365 and adjusted *R*^2^ = 0.289. Model 3: β coefficient = −0.434 and adjusted *R*^2^ = 0.354). Age was also independently associated with anterior labral length in two models (Model 1: β coefficient = −0.423 and adjusted *R*^2^ = 0.303. Model 3: β coefficient = −0.308 and adjusted *R*^2^ = 0.354). Total JHEQ score was independently associated with anterior labral length in two models (Model 2: β coefficient = −0.399 and adjusted *R*^2^ = 0.289. Model 3: β coefficient = −0.273 and adjusted *R*^2^ = 0.354).

**Table VIII.** Univariate and multivariate regression analyses for anterior labral length

	*Univariate analysis*		*Multivariate analysis*					
			*Model 1*		*Model 2*		*Model 3*	
			*Adjusted R^2^ = 0.303*		*Adjusted R^2^ = 0.289*		*Adjusted R^2^ = 0.354*	
	*β (95% CI)*	*P-value*	*β (95% CI)*	*P-value*	*β (95% CI)*	*P-value*	*β (95% CI)*	*P-value*
Patient characteristics								
Age	0.332 (0.038, 0.342)	0.015*	0.423 (0.107, 0.378)	0.001*	–	–	0.308 (0.032, 0.320)	0.017*
Height	0.175 (−0.118, 0.525)	0.209	–	–	–	–	–	–
Weight	0.200 (−0.044, 0.278)	0.151	–	–	–	–	–	–
BMI	0.160 (−0.198, 0.734)	0.254	–	–	–	–	–	–
Bone morphologic parameter								
LCEA	−0.396 (−0.604, −0.128)	0.003*	–	–	–	–	–	–
AHI	−0.397 (−54.919, −11.681)	0.003*	−0.478 (−59.858, −20.176)	0.0001*	−0.365 (−50.340, −10.382)	0.003*	−0.434 (−55.738, −16.924)	0.0001*
Sharp angle	0.187 (−0.175, 0.914)	0.179	–	–	–	–	–	–
ARO	0.394 (0.131, 0.629)	0.004*	–	–	–	–	–	–
FEAR index	0.387 (−0.089, 0.558)	0.008*	–	–	–	–	–	–
JHEQ score								
Pain	−0.35 (−0.578, −0.068)	0.014*	–	–	–	–	–	–
Movement	−0.379 (−0.449, −0.084)	0.005*	–	–	–	–	–	–
Mental	−0.381 (−0.582, 0.110)	0.005*	–	–	–	–	–	–
Total	−0.428 (−0.224, −0.057)	0.001*	–	–	−0.399 (−0.208, −0.053)	0.001*	−0.273 (−0.170, 0.008)	0.031*

Model 1 = Age and AHI.

Model 2 = AHI and pain.

Model 3 = Age, AHI and total JHEQ score.

* Indicates significance (*P* < 0.05).

## DISCUSSION

Even though acetabular bone morphology is certainly important in understanding acetabular dysplasia, little is known about soft tissue abnormalities associated with acetabular dysplasia. More importantly, the relationship between acetabular labral length and patient symptoms remains unknown. In this study, we investigated the relationships among bone morphologic parameters, labral length and symptoms in patients with symptomatic acetabular dysplasia before RAO using preoperative radial MRI. We found that anterior labral length was independently related to total JHEQ score and pain subscale score. Our results suggest that labral length may be an important objective image finding that can be used to assess the severity of cumulative hip instability in clinical practice.

Our results suggest that labral length may be an important objective image finding that can be used to assess the severity of cumulative hip instability. We believe that the surgeon should pay attention to anterior labral length more than bone morphology as an indicator of symptom severity in patients with symptomatic acetabular dysplasia. Kamenaga *et al*. reported that labral length was greater in symptomatic hips (9.5 ± 3.0 mm) than in asymptomatic hips (7.9 ± 2.1 mm) among patients who were diagnosed hip labral tear, femoroacetabular impingement and DDH. Moreover, focusing on borderline DDH, 90.9% of symptomatic subjects had an average labral length of 10 mm or more [21]; in contrast, bone morphology was not related to JHEQ scores. Our findings are in agreement with previous study. Takegami *et al*. reported that only motor score correlated with CEA, which is consistent with the weak relationship with bone morphology in our study (β coefficient = −1.72 and adjusted *R*^2^ = 0.12) [26].

**Fig. 4.**
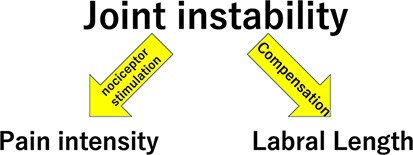
Relationship between labrum and pain caused by hip instability.

We think that a mechanism to compensate for hip instability exists in the labrum of patients with symptomatic acetabular dysplasia (Fig. 4). Although instability was not quantitatively measured in our study, data demonstrated that age was independently related to anterior labral length (β = 0.423 and *P* = 0.001), which suggests that cumulative stress due to hip instability might lead to labral lengthening. In general, the surrounding soft tissue can modify in response to joint instability. For example, in lumbar spondylolisthesis, the longitudinal ligaments and facet capsule become thickened. This soft tissue thickening is considered a response to lumbar vertebral instability. We presume that the same physiological response can occur in the hip joint, where stability is determined not only by bone morphology but also by the state of the soft tissues. Several studies have suggested soft tissue compensatory mechanisms in the presence of hip instability [9, 27]. Horii *et al*. reported that anterior labral length was greater in patients with DDH than in normal patients. Bouthillier *et al*. reported that the superior capsule thickness/femoral width ratio (a measure of capsule thickness) is significantly greater in DDH patients than in patients with an isolated labral tear group (0.24 versus 0.15, *P* < 0.05). It might be important to assess those soft tissue conditions before performing arthroscopic procedure in symptomatic hip, since the hip instability due to acetabular dysplasia is considered as a factor of poor prognosis in hip arthroscopy [28–30].

Although our study demonstrated that anterior labral length is independently related to patient symptoms, further investigation of other factors that may contribute is warranted. First, from an anatomical point of view, the labrum, capsule, ligament teres, surrounding muscles and synovium are soft tissues that contain pain receptors [11, 31, 32]. Second, not only labral length but also labral injury can contribute to hip pain. Santori *et al*. reported that 67% of postoperative patients were satisfied with limbectomy; among these, the mean preoperative modified Harris Hip Score improved from 48.4 to 89.8 three and a half years after surgery [33]. Third and most importantly, the extent of instability can greatly contribute to hip pain. Although a gold standard quantitative method has not been established, several studies have proposed new methodologies, such as MRI in a specific hip position. Akiyama *et al*. [34] performed MRI in four different positions: neutral, 45° flexion, 15° extension and the Patrick position. They reported a significant difference in translation from the neutral position to the Patrick position between normal female hips and dysplastic hips (3.23 ± 0.73 mm versus 4.10 ± 1.41 mm, *P* = 0.016). Future quantitative evaluation of hip instability is warranted.

In the present study, the correlation coefficient was worse for labral index compared with labral length. In contrast, Curley *et al*. reported normalized evaluation for labral size relative to the femoral head diameter [23]. They mentioned that the labral index may be a useful metric to evaluate the labral length in patients with femoroacetabular impingement [23]. This discrepancy may be explained by the following reason. In the femoroacetabular impingement, the variation of femoral head size is minimum. Instead, in acetabular dysplasia, the femoral head size varies according to the acetabular morphology.

Unlike our study, some reports have shown a correlation between bone morphology and patient-reported outcome measures (PROMs); Ibrahim *et al*. showed a correlation between Hip disability and Osteoarthritis Outcome (HOOS) and the posterior coverage of the femoral head before Periacetabular Osteotomy (PAO) [35]. Ibrahim *et al*. also studied the impact on functional outcome after arthroscopic cam resection in patients with symptomatic cam femoroacetabular impingement and reported that only preoperative anterior coverage was negatively correlated with improvement in the symptom subscale of the HOOS [36]. We believe that this discrepancy is that: the first, their two studies were targeted on several hip disorders such as femoroacetabular impingement, not only acetabular dysplasia. The second, it compared the amount of pre- and postoperative changes in PROMs. In contrast, our study focused on only preoperative PROMs.

This study has several limitations. First, because it only included symptomatic patients who required surgery, our findings may not apply to asymptomatic patients or those with less severe symptoms. Second, only hip radiographs of anteroposterior view were used to evaluate the bone morphology; three-dimensional computed tomography, which can provide more detail and is more reliable at assessing joint fissures [37], was not performed. Azuma *et al*. confirmed the close relationship between acetabular coverage in three-dimensional computed tomography images and the anterior–center–posterior angle by modified inlet view [38]. Third, in the present study, the subjects were almost all females. Moreover, the joint laxity, which is relatively frequently seen in female, was not recorded in this study. Therefore, the relationship between the joint laxity and labral length is not clear. Fourth, relatively higher numbers of exclusion criteria were established in this study. The reason for this is that we aimed to minimize the effect from degenerative changes (age > 60 was excluded) and focus on the hip instability due to acetabular dysplasia (LCEA > 25 was excluded). Moreover, the total JHEQ score is affected from the other side. Thereby, if the non-Ope side is worse than the Ope side, the bias would occur. Therefore, we exclude worse JHEQ score on the non-Ope side.

## CONCLUSION

Labral length, notably in anterosuperior area, as measured by radial MRI in symptomatic patients with acetabular dysplasia was related to patient’s symptom.

## Data Availability

The data underlying this article will be made available upon reasonable request to the corresponding author.
